# Functional, Antioxidant, and Antimicrobial Profile of Medicinal Leaves from the Amazon

**DOI:** 10.3390/antiox14080965

**Published:** 2025-08-05

**Authors:** Gabriela Méndez, Elena Coyago-Cruz, Paola Lomas, Marco Cerna, Jorge Heredia-Moya

**Affiliations:** 1 Carrera de Ingeniería en Biotecnología, Universidad Politécnica Salesiana, Sede Quito, Campus El Girón, Av. 12 de Octubre N2422 y Wilson, Quito 170143, Ecuador; 2 Maestría en Productos Farmacéuticos Naturales, Universidad Politécnica Salesiana, Sede Quito, Campus El Girón, Av. 12 de Octubre N2422 y Wilson, Quito 170109, Ecuador; 3 Facultad de Ciencias de la Salud Eugenio Espejo, Centro de Investigación Biomédica (CENBIO), Universidad UTE, Quito 170527, Ecuador

**Keywords:** bioactive compounds, functional food, microextraction, ABTS, DPPH, well diffusion

## Abstract

The Amazon region is home to a remarkable diversity of plant species that are used in traditional medicine and cuisine. This study aimed to evaluate the functional, antioxidant, and antimicrobial properties of the leaves of *Allium schoenoprasum*, *Brugmansia candida* (white and pink), and *Cyclanthemum bipartitum*. Bioactive compounds (*L*-ascorbic acid, organic acids, carotenoids, phenolic compounds, and chlorophylls) were quantified using liquid chromatography. The ABTS and DPPH methods were used to assess the antioxidant capacity. Additionally, the antimicrobial activity against Escherichia coli, Staphylococcus aureus, Pseudomonas aeruginosa, Streptococcus mutans, Candida albicans, and Candida tropicalis was evaluated. The results revealed a high content of *L*-ascorbic acid (7.6 mg/100 g dry weight) and total carotenoids (509.0 mg/100 g dry weight), as well as high antioxidant capacity (4.5 mmol TE/100 g dry weight) and broad antimicrobial activity in Brugmansia candida ‘pink’. The White variety had the highest concentration of total chlorophylls (1742.8 mg/100 g DW), *Cyclanthemum bipartitum* had the highest total organic acid content (2814.5 mg/100 g DW), and *Allium schoenoprasum* had the highest concentration of total phenolic compounds (11,351.6 mg/100 g DW). These results constitute a starting point for future research, emphasizing the potential health risks that certain species may pose.

## 1. Introduction

The Amazon is characterised by high levels of biological diversity, which is not entirely endemic and is the result of a complex evolutionary history [1]. This region is home to plant species that indigenous communities use for food and medicinal purposes. A recent study identified over 220 edible plant species native to the Amazon, demonstrating the region’s diverse plant resources and its potential to contribute to agricultural diversification, strengthen local food systems, and enhance the nutritional outcomes of Amazonian populations [2].

Traditionally, Amazonian medicinal plants have been used to treat various diseases, and this practice has been maintained through the intergenerational transmission of knowledge within indigenous communities. This empirical knowledge, accumulated over centuries, is an important knowledge base on the therapeutic potential of Amazonian plant biodiversity. However, more research is needed to elucidate its practical application. Thus, leaves are among the most widely used plant parts. They are prepared as macerates, infusions, or topical applications to treat conditions such as pain, diarrhoea, skin infections, inflammation, and digestive disorders [3]. These uses are important not only because they are associated with efficacy in traditional use, but also because they are accessible and rooted in traditional medical practices [4].

In this context, several studies on Amazonian plants have reported the presence of bioactive compounds, including flavonoids, terpenoids, saponins, and alkaloids. Plants produce these secondary metabolites as part of their defence mechanisms against biotic and abiotic agents, and have significant pharmacological properties. Phenolic compounds have been documented for their antioxidant capacity, which is associated with protective effects against chronic non-communicable diseases, such as diabetes, cardiovascular disease, neurodegenerative processes, and cancer [5].

Various studies of Amazonian plants have demonstrated therapeutic potential in the treatment of metabolic diseases. For example, a systematic review identified 77 Amazonian plant species that are traditionally used to treat type 2 diabetes mellitus. Of these, approximately 50% showed evidence of hypoglycaemic activity in preclinical trials, which provides scientific basis for their traditional use [6]. The increased research attention in Amazonian plant species has prompted numerous investigations into their antimicrobial properties, particularly in response to the urgent need for natural alternatives amidst rising bacterial resistance. Hydroalcoholic extracts derived from Amazonian leaves have exhibited inhibitory effects against pathogens such as *Staphylococcus aureus*, *Bacillus subtilis*, *Escherichia coli*, and *Pseudomonas aeruginosa*. These findings highlight the potential of these plant species as sources of phytotherapeutic agents with antibacterial efficacy [7].

Among the studied species, *Allium schoenoprasum*, commonly known as chives, has received particular attention for its medicinal value, despite not being native to the Amazon. The leaves of this plant are rich in bioactive phytochemicals, including phenolics, flavonoids, and saponins, which contribute to its antioxidant, anti-inflammatory, antifungal, and anticancer properties. Widely used in culinary practices, chive leaves also demonstrate significant antioxidant activity by reducing cellular oxidative damage at the cellular level [8,9,10]. Furthermore, antifungal assays have demonstrated that their extracts exhibit activity comparable to that of conventional agents, such as fluconazole, when tested against phytopathogenic fungi [11]. Recent ethnopharmacological research continues to document the antimicrobial effects of Amazonian species traditionally used by Indigenous communities. These studies not only validate ancestral knowledge, but also offer potential pathways for the development of plant-based therapies targeting both bacterial and fungal pathogens [12,13].

One such example is *Cyclanthemum bipartitum* (family Cyclanthaceae), a lesser-known Amazonian plant with limited research available, underscoring the research opportunities of the region’s floristic diversity [14]. Another species, *Brugmansia candida*, native to South America, produces tropane alkaloids such as hyoscyamine, scopolamine, and anisodamine, compounds with potent anticholinergic properties that have been employed in the treatment of neurological, digestive, and respiratory disorders [15].

Beyond the Amazon basin, traditional medicinal systems remain central to primary healthcare in rural and Indigenous regions across Mesoamerica. Plants such as *Psidium guajava*, commonly used for gastrointestinal ailments, and *Tagetes lucida*, applied in the management of neurological conditions, form an important component of local healing practices. However, despite their therapeutic use, many of these species still lack comprehensive toxicological and clinical evaluations to ensure safe integration into formal healthcare [16].

Although the pharmacological promise of Amazonian leaves is undeniable, several barriers hinder their adoption in modern medicine. Chief among these is the scarcity of robust, interdisciplinary studies capable of establishing standardised protocols for efficacy, dosage, and safety. Equally important is the need to safeguard ecological sustainability; unsupervised harvesting could threaten plant populations and disturb the ecological balance of Amazonian ecosystems [17,18]. In this context, the present study aimed to explore the phytochemical properties, functional attributes, antioxidant potential, and antimicrobial activity of Amazonian medicinal leaves, to bridge the gap between scientific knowledge and ancestral wisdom.

## 2. Materials and Methods

### 2.1. Reagents and Standards

The analytical grade reagents used in this research were acetone (CAS 67-64-1), fluconazole (CAS 86386-73-4), ABTS (2,2′-azino-bis-(3-ethylbenzothiazoline-6-sulfonic acid) (CAS 30931-67-0), DPPH (2,2-diphenyl-1-picrylhydrazine (CAS 1898-66-4), formic acid (CAS 64-18-6), Folin–Ciocalteu (CAS 7732-18-5), metaphosphoric acid (CAS 37267-86-0), nitric acid (CAS 7697-37-2), potassium chloride (CAS 7447-40.7), potassium persulfate (CAS 7727-21-1), sodium acetate trihydrate (CAS 6131-90.4), sodium carbonate (CAS 497-19-8), and sodium hydroxide (CAS 1310-73-2), all purchased from Sigma-Merck (Darmstadt, Germany). Analytical-grade hydrochloric acid (CAS 7647-01-0) was from Labscan (RCI Labscan, Dublin, Republic of Ireland). Culture media, such as brain–heart infusion (BHI), Mueller–Hinton agar (MHA), and Sabouraud dextrose agar (SDA), were obtained from BD DifcoTM (Fisher Scientific Inc., Madrid, Spain). Dextrose–yeast peptone broth (YPDB) was obtained from SRL (Sisco Research Laboratories Pvt. Ltd., Bombay, India), and streptomycin sulfate (CAS 3810-74-0) from Phytotech (PhytoTechnology Laboratories^®^, Lenexa, KS, USA). In addition, the chromatographic-grade reagents were acetonitrile (CAS 75-05-8), ethanol (CAS 64-17-5), and methanol (CAS 67-56-1), all purchased from Fisher Chemical (Fisher Scientific Inc., Madrid, Spain). The water used during the study was purified with a NANOpure Diamond system (Barnstead Inc., Dubuque, IA, USA).

Analytical standards included β-carotene (93.0%, CAS 7235-40-7), caffeic acid (98.0%, CAS 331-39-5), chlorogenic acid (95.0%, CAS 327-97-9), chrysin (97.0%, CAS 480-40-0), ferulic acid (100.0%, CAS 1135-24-6), and gallic acid (100.0%, CAS 149-91-7). Other standards included 2,5-dihydroxybenzoic acid (98.0%, CAS 490-79-9), 3-hydroxybenzoic acid (99.0%, CAS 99-06-3), kaempferol (97.0%, CAS 520-18-3), luteolin (98%, CAS 491-70-3), *m*-coumaric acid (99.0%, CAS 588-30-7), naringin (95.0%, CAS 10236-47-2), pure *o*-coumaric acid (97.0%, CAS 614-60-8), *p*-coumaric acid (98.0%, CAS 501-98-4), *p*-hydroxybenzoic acid (99.0%, CAS 99-06-3), quercetin (95.0%, CAS 849061-97-8), rutin (94.0%, CAS 153-18-4), shikimic acid (99.0%, CAS 138-59-0), syringic acid (95.0%, CAS 530-57-4), vanillic acid (97.0%, CAS 121-34-6), and trolox (98%, CAS 53188-07-1); all of these standards were purchased from Sigma-Merck (Darmstadt, Germany). In turn, calcium (CAS 7440-70-2), iron (CAS 7439-89-6), magnesium (CAS 7439-95-4), potassium (CAS 7440-09-7), and sodium (CAS 7440-23-5) were purchased from Accustandard (AccuStandard, Inc., New Haven, CT, USA) at a concentration of 1000 μg/mL. The strains used in the study were *Candida albicans* (ATCC 1031), Candida tropicalis (ATCC 13803), *Escherichia coli* (ATCC 8739), *Pseudomonas aeruginosa* (ATCC 9027), *Staphylococcus aureus* (ATCC 6538P), and *Streptococcus mutans* (ATCC 25175), all obtained from the American Type Culture Collection (ATCC) in Manassas, VA, USA.

### 2.2. Physico-Chemical Analyses

**Figure 1:**
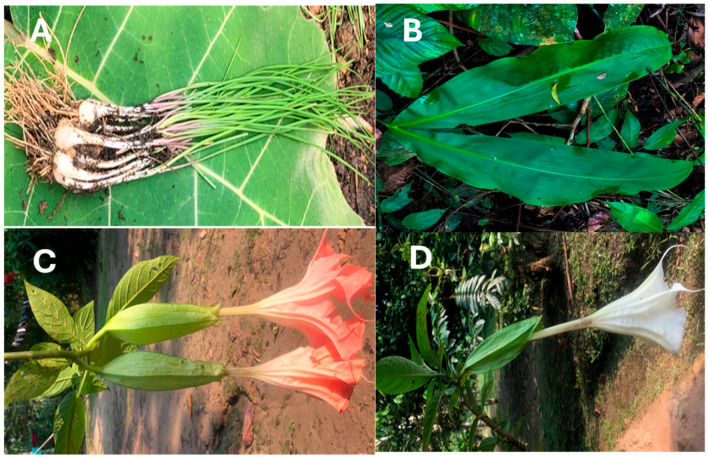
Photograph of the leaves. (**A**) *Allium schoenoprasum* L. (4765, Herbarium QUPS-Ecuador); (**B**), *Cyclanthus bipartitus* Poit. Ex A. Rich. (4776, Herbarium QUPS-Ecuador); (**C**), *Brugmansia candida* Pers. var. pink (4793, Herbarium QUPS-Ecuador); (**D**); *Brugmansia candida* Pers. var. White (4794, Herbarium QUPS-Ecuador).

This study was conducted using four leaves collected in the Amazon region of Ecuador due to their culinary and medicinal significance (Figure 1). To facilitate botanical identification, the plant material was pressed and dried. This was carried out in the herbarium of the Salesian Polytechnic University in Quito-Ecuador.

Fresh leaves were collected at random within the same sector for chemical analysis. At the same time, freeze-dried material was used to analyse bioactive compounds, antimicrobial activity, and antioxidant activity in a Christ Alpha 1-4 LDplus (GmbH, Osterode am Harz, Germany) after freezing at −80 °C. The freeze-dried material was ground, and stored in glass jars in a nitrogen atmosphere at −20 °C until analysis. The physico-chemical characterisation included the quantification of soluble solids, pH, titratable acidity, moisture content, ash content, and minerals. The pH was measured using a SevenMulti S47 (Mettler Toledo, Columbus, OH, USA). Soluble solids were determined using an Hitech RHB-32 refractometer (G-Won Hitech Co., Ltd., Seoul, Republic of Korea), and total titratable acidity was evaluated by acid–base titration. Moisture content was calculated after drying at 100 °C in a Memmert Be 20 oven (Memmert GmbH + Co., KG, Barcelona, Spain). In contrast, ash content was determined by calcination in a muffle furnace (Thermo Fisher Scientific, Waltham, MA, USA) at 550 °C [12,19].

#### 2.2.1. Mineral Profile

For mineral extraction, 40 mg of freeze-dried powder was weighed in a Speed-180 wave Xpert microwave Teflon digestor (Berghof Products + Instruments GmbH, Eningen unter Achalm, Germany) and mixed with 5 mL of 65% nitric acid. A 10 min resting period was followed before sealing the digester. Digestion was carried out with a temperature and pressure gradient defined by the equipment. The samples were cooled to room temperature for 20 min and diluted to a final volume of 25 mL. The quantification of minerals, including calcium, iron, sodium, potassium, and magnesium, was carried out using a Varian SpectrAA-55 atomic absorption spectrophotometer (Varian Inc., Palo Alto, CA, USA), employing specific wavelengths and calibrations. The mineral concentrations were expressed in milligrams per 100 g of dry weight (mg/100 g DW) [12].

#### 2.2.2. Phytochemical Screening

The phytochemical screening involved the analysis of steroids, terpenoids, phenols, tannins, alkaloids, flavonoids, anthraquinones, saponins, and acetoginins, as described in the methodology of León-Fernández et al. [20]. Then, 200 mg of the lyophilised sample was mixed with 1 mL of deionised water, homogenised in a VM-300 vortex (Interbiolab Inc., Orlando, FL, USA), and shaken for 3 min in an FS60 ultrasonic (Fisher Scientific Inc., Waltham, MA, USA). The solid residue was separated by centrifugation, and the aqueous extract was recovered. The solid was extracted twice with 500 µL of water. The supernatants were combined and stored frozen until analysis.

### 2.3. Analysis of Bioactive Compounds

#### 2.3.1. *L*-Ascorbic Acid

*L*-ascorbic acid was quantified by the protocol outlined by Coyago et al. [21]. Twenty milligrams of lyophilised powder were mixed with 1.2 millilitres of 3% metaphosphoric acid and 200 µL of 0.2% homocysteine. This was then homogenised using a VM-300 vortex mixer (Interbiolab Inc., Orlando, FL, USA) and stirred using an FS60 ultrasonicator (Fisher Scientific Inc., Waltham, MA, USA). This was then added to 600 mL of Milli-Q water, filtered and analysed using an RRLC 1200 high-resolution liquid chromatograph (Agilent Technologies, Mississauga, ON, Canada) coupled with a DAD-UV-VIS detector and a ZORBAX Eclipse XDB-C18 column (1.8 µm, 4.6 mm × 50 mm) (Agilent Technologies, Santa Clara, CA, USA). An ascorbic acid standard was used, and the concentration was expressed as milligrams of ascorbic acid per 100 g of dry weight (mg/100 g DW).

#### 2.3.2. Organic Acid Profile

The individual organic acids were determined as described by Coyago et al. [12]. Twenty milligrams of freeze-dried powder were combined with 1.5 mL of 0.02 N sulfuric acid, which included 0.05% metaphosphoric acid and 0.02% homocysteine. The mixture was homogenised using a VM-300 vortex mixer (Interbiolab Inc., Orlando, FL, USA) and then shaken in an FS60 ultrasonic bath (Fisher Scientific Inc., Waltham, MA, USA). The liquid extract was then filtered and quantified using a 1200 RRLC liquid chromatograph (Agilent Technologies, Mississauga, ON, Canada), which was equipped with a DAD-UV-VIS detector and a YMC-Triart C18 column (3 µm, 4.6 mm × 150 mm) (YMC Europe GmbH, Dinslaken, Germany). Standards of citric, tartaric and malic acid were employed for quantification. The concentrations of organic acid were reported in milligrams per 100 g of dry weight (mg/100 g DW).

#### 2.3.3. Carotenoid Profile

The quantification of individual carotenoids was performed in alignment with the protocol delineated by Coyago et al. [13]. Twenty milligrams of freeze-dried powder were combined with 250 µL of methanol, 500 µL of chloroform and 250 µL of Milli-Q water. This solution was subsequently homogenised using a VM-300 vortex mixer (Interbiolab Inc., Orlando, FL, USA) and stirred with an FS60 ultrasonicator (Fisher Scientific Inc., Waltham, MA, USA). This extraction methodology was repeated until the solid matrix was completely decolourised. The coloured phase was then collected and subjected to evaporation to dryness under diminished pressure using a rotary vacuum evaporator at temperatures below 30 °C. To quantify the carotenoids, the dry extract was reconstituted in 20 µL of ethyl acetate and analysed utilising an RRLC 1200 liquid chromatograph (Agilent Technologies, Mississauga, ON, Canada) that was equipped with a DAD-UV-VIS detector and a YMC C30 column (3 µm, 4.6 × 150 mm) (YMC Europe GmbH, Dinslaken, Germany). Reference standards of astaxanthin, α-carotene, β-carotene, β-cryptoxanthin, lycopene, lutein, trans-β-apo-8-carotenal, violaxanthin, and zeaxanthin were employed for identification and quantification. The aggregate carotenoid content was calculated as the sum of the individual carotenoids, expressed in milligrams per 100 g of dry weight (mg/100 g DW).

#### 2.3.4. Phenol Profile

The quantification of individual phenolic compounds was conducted using the methodology established by Coyago et al. [22]. In summary, twenty milligrams of freeze-dried powder were combined with 500 μL of 80% methanol acidified with 0.1% HCl. The mixture was then subjected to homogenisation using a VM-300 vortex (Interbiolab Inc., Orlando, FL, USA) and stirred in an FS60 ultrasonicator (Fisher Scientific Inc., Waltham, MA, USA). The process was repeated on three occasions, after which the methanol phase was recovered and quantified using an RRLC 1200 liquid chromatograph (Agilent Technologies, Mississauga, ON, Canada) equipped with a DAD-UV-VIS detector and a ZORBAX Eclipse Plus C18 column (5 μm, 4.6 mm, × 150 mm) (Agilent Scientific Instruments, Santa Clara, CA, USA). The quantification of phenolic compounds was executed utilising reference standards that include shikimic acid, *p*-hydroxybenzoic acid, 3-hydroxybenzoic acid, 2-methoxybenzoic acid, 3-methoxybenzoic acid, 2,5-dihydroxybenzoic acid, gallic acid, protocatechuic acid, vanillic acid, syringic acid, ellagic acid, tannic acid, *p*-coumaric acid, *m*-coumaric acid and *o*-coumaric acid, chlorogenic, caffeic and ferulic, rutin, quercetin, myricetin, kaempferol, quercetin glucoside, chrysin, luteolin and rutin, catechin and epicatechin, and naringenin and naringin. The assessment of total phenolic content was performed by summing the individual phenolic compounds which were quantified in milligrams of phenolic compound per 100 g of dry weight (mg/100 g dry weight).

### 2.4. Antioxidant Activity Analyses

The antioxidant activity was determined using the DPPH and ABTS methods, as described by Coyago et al. [12]. Twenty milligrams of freeze-dried powder were combined with 2 mL of methanol. Subsequently, the resultant mixture was homogenised in a VM-300 Vortex (Interbiolab Inc., Orlando, FL, USA) and subjected to agitation in an FS60 ultrasonic bath (Fisher Scientific Inc., Waltham, MA, USA). The liquid phase was then exposed to reaction with ABTS•+ or DPPH− radicals, and the ensuing colourimetric changes were quantified using a Multiskan GO microplate reader spectrophotometer (Agilent Scientific Instruments, Santa Clara, CA, USA).

The ABTS radical was prepared by mixing a solution of 7 mM ABTS with 2.45 mM potassium persulphate in a 1:1 ratio and incubating for 16 h. Subsequently, the radical was diluted until the value of the absorption was 0.7 at 754 nm. To quantify the antioxidant activity, 10 µL of the extract or standard was placed with 200 µL of the ABTS radical in a 96-well microplate, following which the reaction was read at 270 nm. The DPPH radical was prepared by accurately weighing 10 mg of the reagent and subsequently diluting it in 50 mL of HPLC-grade methanol. Quantification was performed by mixing 20 µL of the sample or standard with 280 µL of the DPPH radical in a 96-well microplate. The reaction was permitted to proceed for 30 min, after which the solution was measured at a wavelength of 560 nm. A 2.5 nM Trolox standard was used for quantification, with the antioxidant activity expressed in millimoles of Trolox equivalents per 100 g of dry weight (mmol TE/100 g DW).

### 2.5. Antimicrobial Activity Analyses

#### 2.5.1. Antibacterial Activity

For the antibacterial assay, 2 g of freeze-dried powder was combined with 10 mL of 50% ethanol, followed by sequential evaporation through lyophilisation. The resulting dry extract was then reconstituted in 1 mL of sterile distilled water to evaluate antimicrobial activity using the well diffusion method, according to the Clinical and Laboratory Standards Institute (CLSI) guidelines with some modifications [12].

The antibacterial efficacy was evaluated against *Staphylococcus aureus* ATCC 6538P, *Escherichia coli* ATCC 8739, *Pseudomonas aeruginosa* ATCC 9027, and *Streptococcus mutans* ATCC 25175. The microorganisms were propagated in the brain–heart infusion (BHI) broth in triplicate and incubated for 48 h at 37 °C under aerobic conditions. After incubation, the cultures were standardised to a 0.5 McFarland standard (approximately 1.5 × 10^8^ CFU/mL) and inoculated onto Petri dishes containing Mueller–Hinton agar. Wells of 6 mm in diameter were created using the tip of a micropipette, into which 80 µL of the extracts were introduced. The plates were then incubated for an additional 48 h at 35 °C. Streptomycin served as a positive control, while distilled water functioned as a negative control. The antimicrobial activity was assessed by measuring the diameters of the inhibition zones in millimetres (mm) [23].

#### 2.5.2. Antifungal Activity

Antifungal activity was evaluated against *Candida albicans* (ATCC 1031) and Candida tropicalis (CC 13803). Yeast was cultured in yeast peptone dextrose broth in triplicate and incubated for 48 h at 30 °C under aerobic conditions. After incubation, the cultures were standardised to a 0.5 McFarland standard (approximately 10^6^ CFU/mL) and inoculated onto Petri dishes containing Sabouraud dextrose agar. Wells with a diameter of 6 mm were created using the base of a micropipette tip, and 80 µL of extracts were added to each well. The plates were incubated for 48 h at 35 °C. Fluconazole was used as the positive control, and distilled water served as the negative control. Antifungal activity was determined by measuring the diameter of the inhibition zones in millimetres (mm) [24].

### 2.6. Statistical Analysis

For the statistical analysis, the software programs Statgraphics Centurion XVII and RStudio 4.3.3 were used. The results are expressed as the mean plus or minus standard deviation. A one-way ANOVA was used. The mean was separated using Tukey’s test with significant differences at 0.05, and Pearson correlations were used at a 95% confidence level to estimate the differences between the leaves of different species. Principal component analysis (PCA) was used to select the variables that most influenced the differences between leaves. In this analysis, all the variables evaluated were compared, including carotenoids, phenolics, and total organic acid; bioactive compounds and antioxidant activity through DPPH; antimicrobial activity; and the complete set of parameters analysed. As the variables had different units of measurement, a standardization process was carried out in which the values were cantered on their mean and scaled to a unit of variance. This ensured that all variables had a mean of 0 and a variance of 1, preventing those with larger magnitudes from dominating the analysis and ensuring an equal contribution of each variable to the model. Additionally, a heat map analysis was conducted to examine the variables under study.

## 3. Results

### 3.1. Physico-Chemical Characteristics

Table 1 presents the average physico-chemical characteristics of the medicinal leaves under study. The results revealed a pH range of 5.4 (*C. biparitus*) to 6.4 (*B. candida* pink). Soluble solids ranged from 1.3 °Brix (*B. candida* pink) to 2.7 °Brix (*A. schoenoprasum*). Total titratable acidity ranged from 0.2% (*C. bipartitus*) to 0.6% (*B. candida* pink). Moisture content ranged from 59.9% (*C. bipartitus*) to 84.9% (*B. candida* pink). Ash content ranged from 0.8% (*A. schoenoprasum*) to 3.2% (*C. bipartitus*). In turn, the samples analysed showed the highest concentration of potassium, ranging from 1881.6 mg/100 g dry weight (DW) (*B. candida* pink) to 2890.1 mg/100 g DW (*B. candida* white).

Table 2 shows the phytochemical screening of the medicinal leaves studied. The results showed the presence of the studied functional groups in most cases. However, there was no activity for steroids and terpenoids in *C. bipartitus*; alkaloids in all the species studied; flavonoids and saponins in *C. bipartitus* and *B. candida* (white and pink); and anthraquinones in *A. schoenoprasum* and *C. bipartitus*.

### 3.2. Analysis of Bioactive Compounds

Table 3 shows the average values for the concentrations of *L*-ascorbic acid, organic acid, carotenoids, chlorophyll and its derivatives, and phenolic compounds. The *L*-ascorbic acid concentration varied between 18.5 mg/100 g DW (*C. bipartitus*) and 608.8 mg/100 g DW (*A. schoenoprasum*). Total organic acid ranged from 185.4 mg/100 g DW (*B. candida* white) to 2128.8 mg/100 g DW (*A. schoenoprasum)*. Total carotenoids ranged from 0.2 mg/100 g DW (*A. schoenoprasum*) to 509.0 mg/100 g DW (*B. candida* pink). The total chlorophyll and its derivatives ranged from 0.5 mg/100 g DW (*A. schoenoprasum*) to 1742.8 mg/100 g DW (*B. candida* white). Total phenols ranged from 832.3 mg/100 g DW (*B. candida* white) to 11,351.6 mg/100 g DW (*A. schoenoprasum*).

### 3.3. Antioxidant Activity Analyses

Table 4 shows the average antioxidant activity values of the samples studied, as determined by the DPPH and ABTS methods. The antioxidant activity values obtained using the DPPH method ranged from 1.0 mmol TE/100 g DW (*C. bipartitus*) to 4.5 mmol TE/100 g DW (*B. candida* pink). Using the ABTS method, the values ranged from 2.6 mmol TE/100 g DW (*C. bipartitus*) to 4.21 mmol TE/100 g DW (*A. schoenoprasum*).

### 3.4. Antimicrobial Activity Analyses

Table 5 shows the average antimicrobial activity values of the medicinal leaves’ extracts studied, while Table 6 shows the minimum inhibitory concentration. The results showed that most of the leaves exhibited activity against the microorganism studied, except for the extracts from *A. schoenoprasum* leaves, which showed no activity. Additionally, *C. bipartitus* was active against *E. coli*, *C. albicans*, and *C. tropicalis*, and *B. candida* white was active against *P. aeruginosa*.

### 3.5. Statistical Analysis

**Figure 2:**
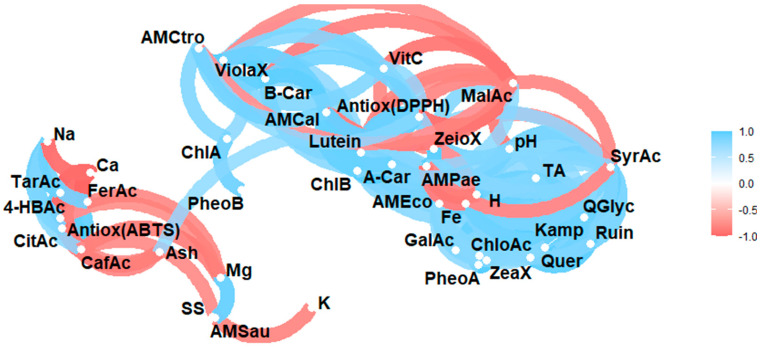
Pearson correlation analysis of the studied variables. Note: Violaxanthin (ViolaX), lutein (Lutein), zeaxanthin (ZeaX), zeionaxanthin (ZeioX), chlorophyll B (ChlB), pheophytin A (PheoA), pheophytin B (PheoB), chlorophyll A (ChlA), A-carotene (A-Car), B-carotene (B-Car), *L*-ascorbic acid (VitC), citric acid (CitAc), malic acid (MalAc), tartaric acid (TarAc), gallic acid (GalAc), 4-hydroxybenzoic acid (4-HBAc), syringic acid (SyrAc), chlorogenic acid (ChloAc), caffeic acid (CafAc), naringenin (Narin), ferulic acid (FerAc), rutin (Ruin), kaempferol (Kamp), quercetin glycoside (QGlyc), quercetin (Quer), calcium (Ca), iron (Fe), potassium (K), magnesium (Mg), sodium (Na), antioxidant activity (Antiox (ABTS) and Antioxidan (DPPH)), antimicrobial activity against *S. aureus* (AMSau), antimicrobial activity against *E. coli* (AMEco), antimicrobial activity against *P. aeruginosa* (AMPae), antimicrobial activity against *C. albicans* (AMCal), and antimicrobial activity against *C. tropicalis* (AMCtro).

Figure 2 shows the results of the Pearson correlation analysis of the studied variables (physico-chemical properties, minerals, *L*-ascorbic acid, organic acids, carotenoids, phenols, and antioxidant and antimicrobial activity).

**Figure 3:**
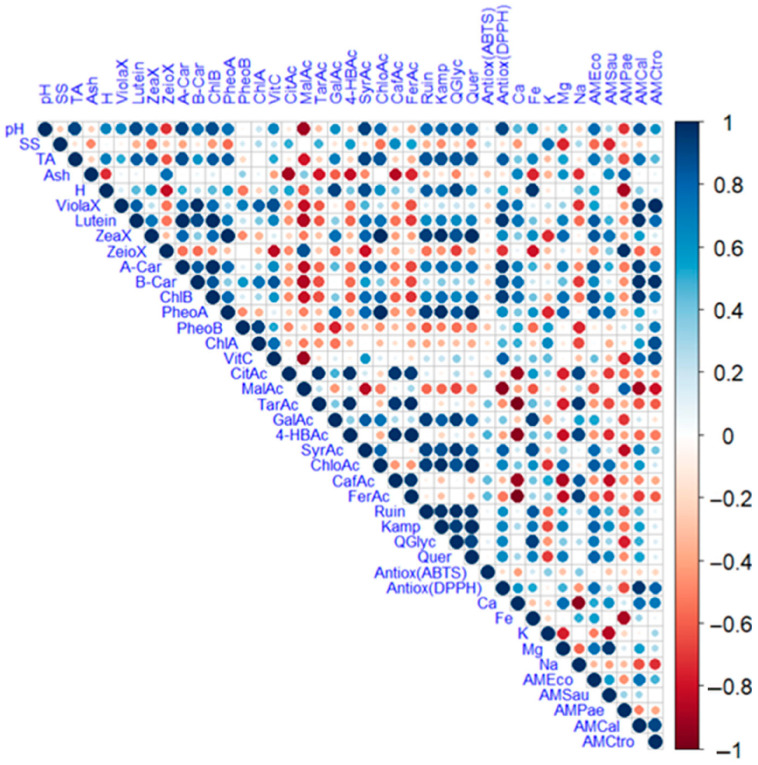
Heat map analysis of the studied variables. Note: violaxanthin (ViolaX), lutein (Lutein), zeaxanthin (ZeaX), zeionaxanthin (ZeioX), chlorophyll B (ChlB), pheophytin A (PheoA), pheophytin B (PheoB), chlorophyll A (ChlA), A-carotene (A-Car), B-carotene (B-Car), *L*-ascorbic acid (VitC), citric acid (CitAc), malic acid (MalAc), tartaric acid (TarAc), gallic acid (GalAc), 4-hydroxybenzoic acid (4-HBAc), syringic acid (SyrAc), chlorogenic acid (ChloAc), caffeic acid (CafAc), naringenin (Narin), ferulic acid (FerAc), rutin (Ruin), kaempferol (Kamp), quercetin glycoside (QGlyc), quercetin (Quer), calcium (Ca), iron (Fe), potassium (K), magnesium (Mg), sodium (Na), antioxidant activity (Antiox (ABTS) and Antioxidan (DPPH)), antimicrobial activity against *S. aureus* (AMSau), antimicrobial activity against *E. coli* (AMEco), antimicrobial activity against *P. aeruginosa* (AMPae), antimicrobial activity against *C. albicans* (AMCal), and antimicrobial activity against *C. tropicalis* (AMCtro).

Figure 3 illustrates a heat map displaying the correlations between the evaluated variables, where blue colours indicate a positive relationship and red colours indicate a negative one. The size of the circles also indicates the level of correlation.

**Figure 4:**
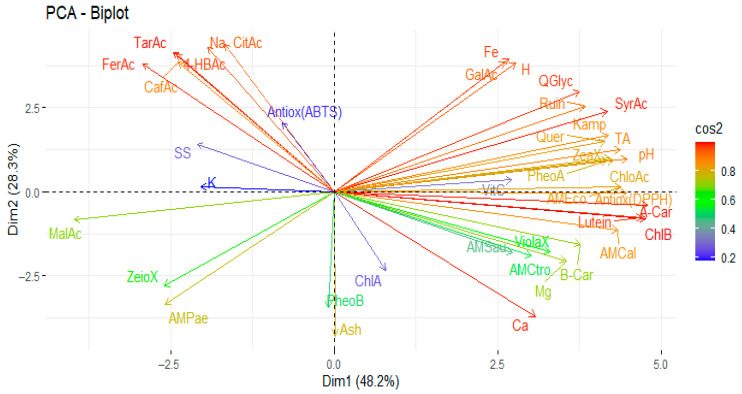
Principal component analysis of the studied variables. Note: violaxanthin (ViolaX), lutein (Lutein), zeaxanthin (ZeaX), zionaxanthin (ZeioX), chlorophyll B (ChlB), pheophytin A (PheoA), pheophytin B (PheoB), chlorophyll A (ChlA), A-carotene (A-Car), B-carotene (B-Car), *L*-ascorbic acid (VitC), citric acid (CitAc), malic acid (MalAc), tartaric acid (TarAc), gallic acid (GalAc), 4-hydroxybenzoic acid (4-HBAc), syringic acid (SyrAc), chlorogenic acid (ChloAc), caffeic acid (CafAc), naringenin (Narin), ferulic acid (FerAc), rutin (Ruin), kaempferol (Kamp), quercetin glycoside (QGlyc), quercetin (Quer), calcium (Ca), iron (Fe), potassium (K), magnesium (Mg), sodium (Na), antioxidant activity (Antiox (ABTS) and Antioxidan (DPPH)), antimicrobial activity against *S. aureus* (AMSau), antimicrobial activity against *E. coli* (AMEco), antimicrobial activity against *P. aeruginosa* (AMPae), antimicrobial activity against *C. albicans* (AMCal), and antimicrobial activity against *C. tropicalis* (AMCtro).

Figure 4 shows the results of the principal component analysis of the studied variables (physico-chemical properties, minerals, *L*-ascorbic acid, organic acids, carotenoids, phenols, and antioxidants and antimicrobial activities).

## 4. Discussion

### 4.1. Physico-Chemical Analyses

The *Brugmansia candida* species, in its white and pink varieties, exhibited the highest pH values (6.0 and 6.4, respectively), indicating a more neutral leaf composition than the other evaluated species. Statistical analysis revealed no significant differences between *Allium schoenoprasum*, *B. candida* white and *B. candida* pink, suggesting that these species are relatively homogeneous in this parameter. This stability may be related to common physiological factors, such as cell structure, organic acid content and leaf maturity. Previous studies have reported high variability in leaf pH among plant species depending on ecological adaptations [25]. For example, certain carnivorous plants have been documented to reach pH values as low as 1. In contrast, *Malvaceae* species can reach values of up to 11, demonstrating the wide chemical plasticity of plant leaves. The pH value obtained for *A. schoenoprasum* in this study was consistent with values reported by other authors, who found a pH of 5.5 in both common and European chives. These similarities may be due to metabolic patterns conserved within the *Allium* genus, which are associated with a content of organic acids and sulphur compounds [26].

Compared to *Cyclanthus bipartitus* and *B. candida* pink, which presented lower values (1.7 and 1.3 °Brix, respectively), *A. schoenoprasum* (2.7 °Brix) and *B, candida* white (2.3 °Brix) showed the highest soluble solids values. Statistical analysis identified two distinct groups: one with a low concentration (*C. bipartitus* and *B. candida* pink) and one with a high concentration, showing statistically significant differences (*p* < 0.05). These variations may be explained by multiple factors, such as species type, photosynthetic metabolism, the type of leaf tissue analysed, and the time of harvest [27]. It has been demonstrated that soluble solids can fluctuate throughout the day and between different plant species. For example, in tropical grasses, these variations are influenced by sugar translocation dynamics [28]. The value found for *A. schoenoprasum* in this study was lower than that reported by other authors (4.65 °Brix for common chives and 5.20 °Brix for European chives), which may reflect agroecological factors such as altitude, light intensity or the phenological stage of the sample [26].

Even more marked differences were observed in titratable acidity. *B. candida* pink had the highest value (0.6%), followed by *B. candida* white (0.4%). In contrast, *C. bipartitus* and *A. schoenoprasum* recorded considerably lower levels. Statistical analysis revealed highly significant differences between all species, indicating substantial variability in the accumulation of organic acids. Other authors explain that this variability may be determined by both the genetic identity of the species and environmental conditions such as soil type, water availability and light stress, which influence the synthesis and accumulation of acidic metabolites [25]. Compared to values reported in the literature, the titratable acidity values in *A. schoenoprasum* were lower than those found by other researchers, who reported values of 0.09 g/100 g in common chives and 0.12 g/100 g in European varieties [26]. This difference may be related to a lower concentration of weak acids in the leaves, possibly influenced by specific growing conditions or the physiological stage of the plant at the time of harvest. Overall, these results suggest that soluble solids and titratable acidity are sensitive parameters that depend on species type and external factors, and that they may determine the sensory and functional profile of the leaves under evaluation.

*B. candida* pink had the highest moisture content (84.9%), followed by *A. schoenoprasum* (80.7%). *C. bipartitus* exhibited the lowest moisture content. These differences can be attributed to the anatomical structure of the leaves, the thickness of the parenchyma, stomatal density and water retention capacity. The moisture content recorded for *A. schoenoprasum* in this study was lower than that reported by other authors, who found values of 88.8% in common varieties and 89.9% in European varieties. This discrepancy could be related to the sampling method, environmental growing conditions, or the physiological state at the time of harvest [26].

In terms of ash content, reflecting total mineral load, *C. bipartitus* had the highest value (3.2%), while *A. schoenoprasum* had the lowest (0.8%). These results suggest that *C. bipartitus* has a greater capacity for mineral accumulation, which may be linked to its physiology being adapted to soils with high ionic availability or to its having efficient mechanisms for mineral absorption and transport.

In terms of their mineral profiles, significant differences were observed between *B. candida* white and pink, which exhibited the highest calcium concentrations (2768.2 and 2620.3 mg/100 g DW, respectively), indicating their potential as valuable sources of this essential mineral. In terms of iron, *B. candida* pink stood out with a concentration of 282.3 mg/100 g dry weight (DW), far exceeding that of the other species. *B. candida* white and *A. schoenoprasum* had the highest potassium values (2890.1 and 2810.3 mg/100 g DW, respectively), reflecting their importance in metabolic functions such as osmoregulation and enzymatic activity. *B. candida* pink also stood out for its high magnesium content (356.4 mg/100 g dry weight), followed by *C. bipartitus* (259.2 mg/100 g dry weight). This could be related to the high chlorophyll content, given that magnesium is a central component of the chlorophyll molecule. Conversely, *A. schoenoprasum* recorded the highest sodium value (20.7 mg/100 g DW), possibly due to its capacity to accumulate sodium in slightly saline environments. Statistical analysis revealed significant differences between species for all evaluated minerals, demonstrating the inherent physiological variability in the absorption, transport and storage capacity of essential nutrients. External factors, such as soil type, pH, water availability, and plant-environment interactions, may also explain these differences. In line with reports by other authors, potassium was found to be the predominant mineral in most species, followed by calcium, a typical pattern in plant tissues due to potassium’s high mobility in sap and its involvement in osmotic and enzymatic processes [29,30].

Qualitative analysis revealed significant differences in the presence of secondary metabolites among the evaluated species. Phenolic compounds, tannins and acetogenins were consistently detected in *A. schoenoprasum*, *C. bipartitus* and both varieties of *B. candida*. This suggests that these metabolites are widely distributed and play an important metabolic role in these plants, possibly in defence mechanisms and antioxidant activity. However, *A. schoenoprasum* stood out for having the most remarkable diversity of metabolites, including steroids, terpenoids, flavonoids, and saponins, indicating a complex phytochemical composition. This metabolic complexity is consistent with previous reports indicating the presence of phenolic compounds, flavonoids and saponins in *Allium* species [8,9,10], thereby reinforcing their functional and pharmacological potential.

By contrast, *B. candida* white and pink share a profile characterised by the presence of steroids, terpenoids, and anthraquinones, though they lack flavonoids and saponins. This composition partially coincides with that reported for the *Brugmansia* genus, which is recognised for its tropane alkaloid and phenolic derivative content [31]. However, no alkaloids were detected in this study. This may be due to the type of plant organ analysed (leaves instead of roots or seeds) or the use of medium-to-high polarity extracts, which may not favour the extraction of alkaloid compounds, which tend to be found in non-polar fractions. Finally, *C. bipartita* had the most limited profile, with only phenols, tannins and acetogenins present. This low diversity may be due to the species’ structural characteristics, its more specialised secondary metabolism, or its lower expression of biosynthetic pathways for other groups of metabolites.

### 4.2. Analysis of Bioactive Compounds

Among the species evaluated, *Brugmansia candida*, both white and pink, had the highest concentrations of *L*-ascorbic acid (7.6 and 5.2 mg/100 g DW, respectively), indicating their potential as natural sources of ascorbic acid. This variation in *L*-ascorbic acid content reflects interspecific variability, which is influenced by genetic factors and environmental conditions. Other authors have demonstrated this in a comparative study of green leafy vegetables, reporting values ranging from 7.2 to 161.0 mg/100 g dry weight (DW) [32]. In this context, the differences observed in the present study may result from the ability of each species to synthesise and accumulate antioxidant compounds, as well as to factors such as degree of exposure to sunlight, soil type, water availability and phenological stage at harvest. In the case of *A. schoenoprasum*, the *L*-ascorbic acid content was lower than that of the *Brugmansia* species. However, these results are consistent with previous studies reporting the presence of ascorbic acid in their leaves [33]. However, the concentrations of *L*-ascorbic acid found in this study are low when compared to the daily recommendations established by international organisations. The US-based Institute of Medicine suggests a consumption of 90 mg/day for men and 75 mg/day for women, while the European Food Safety Authority suggest 110 mg/day for men and 95 mg/day for women [34].

Of the organic acids present in the analysed leaves, malic acid was the most abundant, followed by citric acid and tartaric acid. This distribution is likely associated with the physiological function of malic acid in plants that use the crassulacean acid metabolism (CAM) pathway. In these plants, malic acid plays a crucial role in the nocturnal fixation of CO_2_ and its subsequent use in photosynthesis during the day, thereby optimising water use. During this process, CO_2_ is captured at night and stored in the form of malic acid in the vacuoles. During the day, when the stomata remain closed to minimise water loss, this malic acid is converted back into CO_2_. This metabolic strategy confers an adaptive advantage in arid environments by improving water use efficiency [35]. Quantitatively, *Cyclanthemum bipartitum* had the highest total organic acid content (2814.5 mg/100 g DW), with a particularly high concentration of malic acid (2746.3 mg/100 g DW). This suggests physiological specialisation towards accumulating this metabolite. This was followed by *A. schoenoprasum* (2128.8 mg/100 g DW), which had a high concentration of malic acid and, to a lesser extent, citric acid. There were statistically significant differences between species, indicating high interspecific variability in the biosynthesis and accumulation of organic acids. This variability is influenced by multiple factors, including species genetics, environmental conditions, seasonality, the part of the plant analysed and the extraction and quantification methods used. In addition to their metabolic role, organic acids contribute to the sour taste, aroma and nutritional value of leaves, and also participate in the defence and preservation of plant tissues [21,36,37].

The present study observed various carotenoids in the analysed leaves, including violaxanthin, lutein, zeaxanthin, zeinoxanthin, α-carotene, and β-carotene. Lutein and β-carotene were the dominant compounds. The accumulation of these pigments is regulated by the carotenoid biosynthetic pathway, which involves specific enzymes, including phytoene synthase, lycopene β-cyclase, and β-carotene hydroxylase. The expression of these enzymes is modulated by developmental signals and environmental factors, including light intensity and oxidative stress. This allows plants to adjust their pigment content dynamically [35,38]. *Brugmansia candida* pink exhibited the most diverse and concentrated profile, with high levels of lutein (263.8 mg/100 g DW), β-carotene (153.8 mg/100 g DW), and violaxanthin (43.4 mg/100 g DW). The white variety displayed a similar profile, albeit with slightly lower concentrations. In contrast, *C. bipartitum* showed moderate carotenoids levels (12.5 mg/100 g DW), while *A. schoenoprasum* had a negligible amount (0.2 mg/100 g DW). This can be attributed to the lower activity of the biosynthetic pathways in these species. However, when the results are compared with those in the literature, an apparent discrepancy emerges for *A. schoenoprasum*, as other authors have reported total carotenoid concentrations of 6.9 mg/g in common varieties and 6.3 mg/g in European varieties [26]. However, this methodological difference may result from the fact that the study above used UV-vis spectrophotometry, a technique that measures overall pigment absorbance without discriminating between individual compounds. In contrast, the present study employed chromatography, which enabled the specific and accurate quantification of each carotenoid, as suggested by other authors [39]. In the context of health, the leaves of *A. schoenoprasum* and *C. bipartitum* are a significant source of provitamin A, as the European authority recommends an intake of 750 µg of retinol equivalent (RE)/day for men and 650 µg RE/day for women [40].

Chlorophyll a, chlorophyll b, pheophytin a and pheophytin b were detected in the analysed samples, with chlorophyll b predominating in most cases. This pattern can be explained by the chlorophyll interconversion cycle, whereby the enzyme chlorophyllide a oxygenase catalyses the transformation of chlorophyll a into chlorophyll b, favouring the accumulation of the latter under specific environmental conditions such as low light intensity or stressful situations. Indeed, it has been reported that in highly polluted urban environments, the ratio of chlorophyll a to chlorophyll b tends to decrease, reflecting a relative increase in the latter due to alterations in the biosynthesis and degradation of photosynthetic pigments [41].

In quantitative terms, *B. candida* white had the highest total chlorophyll content (1742.8 mg/100 g DW), followed by *Brugmansia candida* pink (1442.6 mg/100 g DW). Both species exhibited high concentrations of chlorophyll a and b, indicating high photosynthetic activity. *C. bipartitum* exhibited an intermediate content (144.8 mg/100 g DW), whereas *A. schoenoprasum* displayed residual levels (0.5 mg/100 g DW). These differences could be due to physiological, anatomical, and environmental factors that affect the synthesis and accumulation of chlorophyll pigments, such as light exposure, leaf age, or chloroplast density.

When compared with the literature, the values found for *A. schoenoprasum* in this study were considerably lower than those reported by other authors. These authors reported chlorophyll a concentrations of 27.6 mg/g in common varieties and 23.8 mg/g in European varieties, as well as chlorophyll b concentrations of 12.4 mg/g and 9.8 mg/g, respectively [26]. This discrepancy may be primarily due to methodological differences, as the study above employed UV-Vis spectrophotometry to measure the overall absorbance of the pigment in methanolic extracts. In contrast, this study employed a more specific chromatographic methodology, accompanied by spectral analysis, to quantify each of the pigments present individually and accurately.

The leaves analysed in the present study showed a diverse profile of phenolic compounds. The presence of gallic acid, 4-hydroxybenzoic acid, syringic acid, chlorogenic acid, caffeic acid, ferulic acid, rutin, kaempferol, quercetin glucoside, and quercetin were identified, with ferulic acid and caffeic acid being the most abundant. The higher concentrations of these two compounds can be explained by their synthesis through the phenylpropanoid pathway, a fundamental metabolic route in plants involved in the production of secondary metabolites that play roles in defence functions, lignification, the regulation of oxidative stress, and environmental adaptation [35,38]. *A. schoenoprasum* had the highest total phenol content (11,351.6 mg/100 g DW), dominated by significant concentrations of ferulic acid (8697.6 mg/100 g DW) and caffeic acid, followed by 4-hydroxybenzoic acid. This demonstrates its rich biosynthetic capacity for functional phenolic compounds. These findings are consistent with previous studies that have documented the presence of phenolic acids, such as ferulic, *p*-coumaric, gallic, vanillic, and sinapic acids, as well as flavonoids, including quercetin, in *A. schoenoprasum* [33]. In the context of health, a daily intake of 1000 mg of ferulic acid over six weeks resulted in a significant improvement in lipid profiles, a reduction in oxidative stress, and a decrease in inflammatory markers [42]. These findings suggest a potential for reducing cardiovascular disease risk factors.

*B. candida* pink had an intermediate total phenolic content of 3854.2 mg/100 g DW. It stood out due to its high concentration of chlorogenic acid (1707.6 mg/100 g) and flavonoids, such as quercetin and rutin. These compounds are widely associated with antioxidant and anti-inflammatory properties. *C. bipartitum* had a more modest profile (1423.1 mg/100 g DW), dominated by ferulic acid (1271.5 mg/100 g DW), which suggests specialisation in this phenolic compound due to protection against environmental stress. Finally, *B. candida* White recorded the lowest value (832.3 mg/100 g DW), with chlorogenic acid and rutin present in lower concentrations. This reflects either lower phenylpropanoid activity or different expression of secondary metabolic regulatory genes.

### 4.3. Antioxidant Activity Analyses

The results obtained for antioxidant activity using the DPPH and ABTS methods showed significant differences between the evaluated species, mainly due to differences in the detection principles of each technique and the chemical nature of the compounds present in the leaves. The DPPH method is susceptible to the presence of hydroxyl groups in phenolic compounds, especially those with conjugated structures, such as flavonoids, which have multiple OH groups. This method is based on the ability of antioxidant compounds to donate an electron or a hydrogen atom to the DPPH free radical. Consequently, this method more accurately reflects the antiradical capacity of lipophilic or semipolar compounds with high reducing power. In contrast, the ABTS assay involves generating the ABTS radical cation, which both hydrophilic and lipophilic antioxidants can reduce. This makes the ABTS assay a more versatile method, as it is less dependent on the specific structure of the antioxidant. This feature enhances its suitability for quantifying the activity of a broader range of compounds, including dihydrochalcones, flavanones, and low-molecular-weight phenolic acids [19,43].

According to the results obtained using the DPPH method, the pink variety of *Brugmansia candida* had the highest antioxidant capacity (4.5 mmol TE/100 g DW), followed by the white variety (4.0 mmol TE/100 g DW). Both varieties had previously shown high concentrations of carotenoids and flavonoids, which supports their high activity against the DPPH radical. *Allium schoenoprasum* exhibited intermediate antioxidant capacity (1.7 mmol TE/100 g DW). In contrast, *Cyclanthemum bipartitum* exhibited the lowest capacity (1.0 mmol TE/100 g DW), consistent with its limited phytochemical diversity and low flavonoid content. However, evaluation using the ABTS method produced different results, with *A. schoenoprasum* exhibiting the highest antioxidant activity (4.1 mmol TE/100 g DW). This can be attributed to its high concentration of water-soluble phenolic compounds, including ferulic acid, caffeic acid, and 4-hydroxybenzoic acid, which react efficiently with the ABTS cation. This was followed by *Brugmansia candida* pink (2.8 mmol TE/100 g DW), *B. candida* white (2.7 mmol TE/100 g DW) and *C. bipartitus* (2.6 mmol TE/100 g DW), with less pronounced differences between them. In the context of health, ferulic acid has been shown to inhibit ferroptosis, a form of cell death associated with iron-dependent lipid peroxidation, by reducing the production of reactive oxygen species (ROS) and iron aggregation [44]. Conversely, the presence of beta-carotene has been demonstrated to effectively quench singlet oxygen, a highly reactive form of oxygen, thereby preventing oxidative damage to lipids and other biomolecules [45].

### 4.4. Antimicrobial Activity Analyses

The results of the agar diffusion test demonstrate significant variability in the antimicrobial activity of leaf extracts across the evaluated species. *Allium schoenoprasum* did not exhibit any inhibition zones against the bacterial strains tested (*E. coli*, *S. aureus*, *P. aeruginosa* and *S. mutans*) or the fungal strains tested (*C. albicans* and *C. tropicalis*). This suggests that the aqueous extract used contains compounds with low or no antimicrobial activity. By contrast, *Cyclanthemum bipartitum* exhibited selective antibacterial activity, demonstrating moderate inhibition zones against both Gram-positive and Gram-negative bacteria; however, no activity was observed against the yeasts tested. *Brugmansia candida* pink exhibited the broadest and most potent antimicrobial spectrum, demonstrating inhibitory activity against all the bacterial and fungal strains tested. Notably, it produced inhibition zones of up to 11.5 mm against *P. aeruginosa* and *C. tropicalis*. While the positive control (a commercial antibiotic) exhibited larger inhibition zones (>22 mm for bacteria and >10 mm for fungi), *B. candida* pink was identified as the most effective plant extract among those analysed.

From a quantitative standpoint, the evaluation of the minimum inhibitory concentration (MIC) confirmed these findings. *B. candida* pink exhibited the lowest MICs against *S. aureus* (10.6 mg/mL), *S. mutans* (5.3 mg/mL), *E. coli* (10.6 mg/mL), and *P. aeruginosa* (21.1 mg/mL), as well as an antifungal MIC of 42.2 mg/mL against *C. albicans* and *C. tropicalis. B. candida* white exhibited a more limited profile, with high minimum inhibitory concentrations (MICs) against bacteria and fungi (41.7–20.8 mg/mL), but did not show activity against *P. aeruginosa*. *C. bipartitus* had the most restricted effect, being active only against *P. aeruginosa* (125.0 mg/mL), *S. aureus* (31.3 mg/mL), and *S. mutans* (2.0 mg/mL), with no detectable activity against *E. coli* or *Candida* species. These differences may be related to the phytochemical composition of each species, as *B. candida* pink exhibits a high content of phenols, flavonoids, acetogenins, and carotenoids, i.e., compounds known for their antimicrobial properties. In contrast, *A. schoenoprasum* showed little diversity of active metabolites in the aqueous extract, which could explain its inactivity. In addition, *B. candida* extract is an important source in the treatment of diseases caused by *S. aureus* (respiratory, skin, and systemic infections), *E. coli* (urinary and intestinal infections), *P. aeruginosa* (opportunistic microorganism), *C. albicans* (candidiasis), and *C. tropicalis* (opportunistic fungus), as suggested by other authors [24,46].

Some studies have reported the antimicrobial activity of *A. schoenoprasum* against *S. aureus* and *E. coli* using concentrated ethanolic extracts [47,48], as detailed in the literature. However, the absence of activity in the present study can be explained by the use of lyophilised extracts that were resuspended in water and contained no traces of ethanol. This eliminates the possibility of false positives being attributed to ethanol, given that even concentrations as low as 5% can inhibit bacterial growth [49]. Furthermore, no previous reports of antimicrobial activity in *C. bipartitum* or *B. candida* were found, so the results of this study provide novel evidence of the antimicrobial potential of these species.

In this context, the antimicrobial activity observed could be influenced by metabolites found in higher proportions in the sample. Ferulic acid, for instance, has been demonstrated to impede biofilm formation in a range of pathogens [50]. Caffeic acid and its derivatives have been reported as agents capable of permeabilising cell membranes, inducing structural alterations and causing the loss of ions such as potassium, which leads to cell death. This mechanism is particularly effective against Gram-positive bacteria, such as *Staphylococcus aureus* [51]. In addition, 4-hydroxybenzoic acid has been documented to have antimicrobial activity against a wide range of microorganisms, including Gram-positive and Gram-negative bacteria and yeasts such as *Candida albicans* [52]. About lutein, its antibacterial effect against strains such as *Escherichia coli* and *Staphylococcus aureus* has been documented. This effect has been attributed, at least in part, to its capacity to modify cell membrane integrity and bacterial morphology, as evidenced in studies with *E. coli* [53].

### 4.5. Statistical Analysis

Analysis of the correlations between the physico-chemical variables, minerals, bioactive metabolites, and antioxidant and antimicrobial activities revealed significant positive associations, suggesting functional relationships between the various compounds and properties. Direct correlations were observed between soluble solids and total titratable acidity, as well as between lutein and pH, and between lutein and titratable acidity. Similarly, zeaxanthin showed a positive correlation with titratable acidity, while α-carotene was significantly related to pH, titratable acidity, and lutein. β-carotene showed a positive correlation with violaxanthin, lutein, and α-carotene, while chlorophyll b exhibited positive associations with pH, lutein, and α-carotene. Pheophytin correlated with zeaxanthin, and chlorophyll with violaxanthin and β-carotene. *L*-ascorbic acid was significantly associated with violaxanthin, β-carotene, and chlorophyll a, while malic acid was correlated with zeinoxanthin, and tartaric acid was correlated with citric acid. Additionally, gallic acid correlated with moisture, zeaxanthin, and phaeophytin A, 4-hydroxybenzoic acid correlated with citric and tartaric acids, and syringic acid correlated with pH, titratable acidity, and moisture. Ferulic acid was associated with citric, tartaric, and 4-hydroxybenzoic acids. A strong association was observed between flavonoids such as rutin, kaempferol, quercetin glycoside, and quercetin, as well as between these and pH, titratable acidity, moisture, lutein, zeaxanthin, α-carotene, chlorophyll b, phaeophytin a, gallic acid, syringic acid, and chlorogenic acid.

The antioxidant activity, as determined by the DPPH method, showed a positive correlation with pH, titratable acidity, violaxanthin, lutein, α-carotene, β-carotene, chlorophyll b, *L*-ascorbic acid and syringic acid. Regarding minerals, iron showed correlations with moisture, gallic acid, syringic acid, rutin, and quercetin glucoside, while potassium correlated with soluble solids. Magnesium correlated with zeaxanthin, chlorophyll b, phaeophytin a, chlorophyll a, and calcium, and sodium correlated with citric acid, tartaric acid, 4-hydroxybenzoic acid, caffeic acid, and ferulic acid. Additionally, associations were found between antimicrobial activity against *E. coli* and various bioactive and antioxidant variables, including pH, titratable acidity, lutein, zeaxanthin, α-carotene, chlorophyll b, phaeophytin a, syringic acid, chlorogenic acid, rutin, kaempferol, quercetin glucoside, quercetin, and DPPH antioxidant activity. Similarly, activity against *S. aureus* was associated with ash, zeaxanthin, phaeophytin A, chlorogenic acid, kaempferol, quercetin, calcium, and magnesium. In contrast, activity against *P. aeruginosa* was correlated with ash, zeinoxanthin and malic acid. *Candida albicans* antimicrobial activity showed positive associations with pH, titratable acidity, violaxanthin, lutein, α-carotene, β-carotene, chlorophyll b, *L*-ascorbic acid, syringic acid, chlorophyll a, kaempferol, quercetin glucoside, quercetin, and DPPH antioxidant activity. There was also a positive association with activity against *E. coli*. Likewise, activity against *C. tropicalis* was related to violaxanthin, lutein, α-carotene, β-carotene, chlorophyll b, phaeophytin b, chlorophyll a, *L*-ascorbic acid, DPPH antioxidant activity, and calcium. These findings are consistent with previous studies reporting positive correlations between carotenoids, particularly violaxanthin and lutein, and antioxidant activity in *Medicago polymorpha* leaves [54], as well as between soluble solids, carotenoids, phenols, saponins, and ash content in *Artocarpus heterophyllus* leaves [37]. Furthermore, correlations have been found between antimicrobial activity against *S. aureus* and the presence of certain compounds, including *p*-coumaric acid, caffeic acid, gallic acid, and chlorogenic acid [55,56].

Significant negative correlations were also identified. Moisture correlated negatively with ash, while zeaxanthin correlated negatively with moisture and titratable acidity. *L*-ascorbic acid correlated negatively with zeaxanthin, and malic acid correlated negatively with pH, titratable acidity, moisture, violaxanthin, lutein, α-carotene, β-carotene, chlorophyll b, and *L*-ascorbic acid. Additionally, tartaric acid was negatively correlated with ash, gallic acid with phaeophytin b, and 4-hydroxybenzoic acid with ash. Ferulic acid showed negative correlations with ash, violaxanthin, lutein, α-carotene, β-carotene, and chlorophyll b. The flavonoids rutin, kaempferol, quercetin glucoside, and quercetin were negatively correlated with zeinoxanthin, phaeophytin B, and malic acid. DPPH antioxidant activity was negatively correlated with malic acid, while calcium showed negative correlations with citric, tartaric, 4-hydroxybenzoic, caffeic, and ferulic acids. Magnesium showed negative correlations with ferulic and potassium acids, and sodium showed a negative correlation with phaeophytin b. Furthermore, antimicrobial activity against S. aureus exhibited negative correlations with soluble solids, citric acid, tartaric acid, caffeic acid, ferulic acid, and potassium. Conversely, activity against *P. aeruginosa* correlated with pH, titratable acidity, moisture, *L*-ascorbic acid, gallic acid, syringic acid, quercetin glucoside, and iron. Activity against *C. albicans* showed negative correlations with malic acid, ferulic acid, and sodium.

A principal component analysis (PCA) was performed, incorporating physico-chemical variables, bioactive compounds, and antioxidant and antimicrobial activities. This analysis revealed that the first two principal components together explained 76.5% of the total variability, with contributions of 48.2% and 28.3%, respectively, from the first and second components. The variables that contributed most to the total variance are shown in red in the graph and include lutein, α-carotene, chlorophyll b, calcium, syringic acid, quercetin glucoside, iron, ferulic acid, and tartaric acid. The antioxidant activity of ABTS was primarily influenced by citric acid, sodium, and 4-hydroxybenzoic acid. Antimicrobial activity against *P. aeruginosa* was primarily attributed to zeaxanthin. In contrast, activity against *C. tropicalis* and *S. aureus* was attributed to violaxanthin, β-carotene, magnesium, and calcium. Finally, the antimicrobial activity against *C. albicans* was observed with β-carotene, lutein, and chlorophyll b. Finally, the antioxidant activity, as measured by the DPPH assay, and the inhibition of *E. coli* were strongly influenced by lutein, α-carotene, chlorophyll b, pheophytin a, chlorogenic acid, *L*-ascorbic acid, and pH.

In this context, while the results obtained with *Brugmansia candida* are encouraging, the presence of toxic compounds that restrict its direct use must be acknowledged. This opens a relevant field of research into the bioavailability, metabolism, and possible synergistic interactions of these metabolites in vivo. Furthermore, while the study reveals notable correlations between bioactive compounds and biological activity, the underlying causal mechanisms are unclear. This makes it crucial to validate them further using more specific biochemical and molecular approaches.

## 5. Conclusions

This study revealed substantial variations in physicochemical composition, bioactive compound content, antioxidant capacity, and antimicrobial activity among the plant species examined. *Brugmansia candida* (the pink variety) was found to have a high concentration of lutein, ascorbic acid, and tartaric acid, which are compounds that have been linked to high antioxidant capacity according to the DPPH method. In addition, it demonstrated significant antimicrobial activity against various strains, including *Escherichia coli*, *Pseudomonas aeruginosa*, *Streptococcus mutans*, *Staphylococcus aureus,* and *Candida tropicalis*. *Allium schoenoprasum* exhibited the highest concentrations of phenolic acids, particularly ferulic acid, caffeic acid, and 4-hydroxybenzoic acid, as evidenced by its notably high antioxidant capacity, as determined by the ABTS method. However, no antimicrobial activity was observed against the strains evaluated. *Cyclanthemum bipartitum*, a plant traditionally used in the Ecuadorian Amazon for culinary purposes, was characterised by its high malic acid and calcium content, as well as antimicrobial activity against *S. mutans*. Multivariate analysis (PCA and correlations) confirmed the metabolic differentiation between species, identifying relevant associations between compounds such as lutein, α-carotene, chlorophyll b, syringic acid, quercetin glucoside, iron and calcium, and the variability in the biological activity observed. Furthermore, an inverse correlation was identified between ash content and phenolic compounds, suggesting the possibility of metabolic competition in the biosynthesis of secondary metabolites. Despite the encouraging bioactive profile exhibited by *B. candida* rosa, caution should be exercised when considering its utilisation, given the established presence of tropane alkaloids, which have been associated with documented toxicity. Consequently, it is imperative to undertake rigorous toxicological studies and in vivo trials to substantiate the safety of its extracts before contemplating any therapeutic or functional applications.

## Figures and Tables

**Table 1:** Average values of the physico-chemical characteristics of the medicinal leaves.

	Allium schoenoprasum	Cyclanthus bipartitus	Brugmansia candida White	Brugmansia candida Pink
pH	5.7 ± 0.2 ^a^	5.4 ± 0.2 ^ab^	6.0 ± 0.6 ^a^	6.4 ± 0.1 ^a^
Soluble solids (°Brix)	2.7 ± 0.6 ^a^	1.7 ± 0.6 ^b^	2.3 ± 0.6 ^a^	1.3 ± 0.6 ^b^
Total titratable acidity (%)	0.3 ± 0.1 ^c^	0.2 ± 0.0 ^d^	0.4 ± 0.0 ^b^	0.6 ± 0.1 ^a^
Humidity (%)	80.7 ± 3.3 ^b^	59.9 ± 3.7 ^d^	70.2 ± 1.9 ^c^	84.9 ± 0.1 ^a^
Ash (%)	0.8 ± 0.1 ^c^	3.2 ± 0.1 ^a^	2.2 ± 0.4 ^b^	2.1 ± 0.2 ^b^
Mineral profile (mg/100 g DW)				
Ca	233.5 ± 6.1 ^d^	2034.7 ± 44.8 ^c^	2768.2 ± 15.5 ^a^	2620.3 ± 19.8 ^b^
Fe	283.3 ± 26.1 ^b^	69.3 ± 5.8 ^d^	169.0 ± 7.2 ^c^	320.0 ± 24.4 ^a^
K	2810.3 ± 34.8 ^a^	2173.2 ± 13.3 ^b^	2890.1 ± 10.7 ^a^	1881.6 ± 15.9 ^c^
Mg	82.9 ± 0.8 ^c^	259.2 ± 0.1 ^b^	205.7 ± 6.6 ^b^	356.4 ± 34.8 ^a^
Na	20.7 ± 0.4 ^a^	12.0 ± 0.7 ^b^	8.8 ± 0.7 ^c^	13.1 ± 0.1 ^b^

Note: The lowercase letters situated adjacent to the standard deviation denote the separation of means with a confidence level of 95%.

**Table 2:** Phytochemical screening of the medicinal leaves.

Metabolite	*Allium* *schoenoprasum*	*Cyclanthus* *bipartitus*	*Brugmansia* *candida* White	*Brugmansia* *candida* Pink
Steroids	+		+	+
Terpenoids	+	−	+	+
Phenols	+	+	+	+
Tannins	+	+	+	+
Alkaloids	−	−	−	−
Flavonoids	+	−	−	−
Anthraquinones	−	−	+	+
Saponins	+	−	−	−
Acetoginins	+	+	+	+

Note: −, negative test result; +, positive test result.

**Table 3:** Average values of the bioactive compounds of the medicinal leaves.

		*Allium schoenoprasum*	*Cyclanthus bipartitus*	*Brugmansia candida* White	*Brugmansia candida* Pink
*L*-ascorbic acid (mg/100 g DW)		4.3 ± 0.1 ^c^	1.4 ± 0.1 ^d^	7.6 ± 0.5 ^a^	5.2 ± 0.0 ^b^
Organic acid profile (mg/100 g DW)					
	Citric acid	608.8 ± 46.3 ^a^	18.5 ± 5.2 ^d^	88.2 ± 2.2 ^c^	126.3 ± 12.2 ^b^
	Malic acid	1323.4 ± 66.4 ^b^	2746.3 ± 28.6 ^a^	68.9 ± 1.7 ^c^	41.7 ± 3.2 ^d^
	Tartaric acid	196.5 ± 3.4 ^a^	49.7 ± 3.1 ^b^	28.3 ± 1.5 ^c^	45.1 ± 3.3 ^b^
	Total organic acid	2128.8 ± 11.6 ^b^	2814.5 ± 29.4 ^a^	185.4 ± 1.1 ^c^	213.1 ± 18.6 ^b^
Carotenoid profile (mg/100 g DW)					
	Violaxanthin			79.2 ± 7.2 ^a^	43.4 ± 1.9 ^b^
	Lutein	0.2 ± 0.0 ^d^	11.1 ± 0.4 ^c^	191.7 ± 1.0 ^b^	263.8 ± 3.7 ^a^
	Zeaxanthin		0.7 ± 0.0 ^b^		42.0 ± 2.2 ^a^
	Zeionaxanthin		0.7 ± 0.0		
	α-carotene			3.8 ± 0.5 ^b^	6.0 ± 0.1 ^a^
	β-carotene			219.2 ± 5.8 ^a^	153.8 ± 6.7 ^b^
	Total carotenoid	0.2 ± 0.0 ^c^	12.5 ± 0.4 ^b^	493.9 ± 14.5 ^a^	509.0 ± 7.2 ^a^
Chlorophylls and their derivatives (mg/100 g DW)					
	Chlorophyll a				1523.28 ± 22.23
	Chlorophyll b	0.4 ± 0.0 ^d^	152.3 ± 5.1 ^c^	1002.4 ± 45.8 ^b^	1523.3 ± 18.1 ^a^
	Pheophytin a		12.1 ± 1.6 ^b^		280.0 ± 1.9 ^a^
	Pheophytin b	0.2 ± 0.0 ^c^	16.6 ± 0.4 ^b^	51.3 ± 7.9 ^a^	
	Total chlorophylls	0.5 ± 0.3 ^d^	144.8 ± 8.1 ^c^	1742.8 ± 53.6 ^a^	1442.6 ± 80.7 ^b^
Total phenols (mg/100 g DW)					
	Galic acid	118.1 ± 1.9 ^b^	19.6 ± 2.6 ^d^	26.3 ± 0.1 ^c^	159.9 ± 2.3 ^a^
	4-hydroxybenzoic acid	754.0 ± 50.0			
	Syringic acid	107.5 ± 8.6 ^b^	26.3 ± 3.9 ^c^	103.8 ± 2.0 ^b^	182.4 ± 0.7 ^a^
	Chlorogenic	67.7 ± 3.4 ^c^	105.8 ± 2.3 ^b^	112.8 ± 1.1 ^b^	1707.6 ± 28.6 ^a^
	Caffeic acid	1194.9 ± 25.9 ^a^		209.5 ± 1.4 ^b^	
	Ferulic acid	8697.6 ± 24.4 ^a^	1271.5 ± 32.2 ^b^	237.9 ± 4.8 ^c^	
	Rutina	126.3 ± 3.1 ^b^		35.3 ± 0.7 ^c^	348.6 ± 4.8 ^a^
	Kamferol	50.0 ± 1.3 ^b^		22.4 ± 0.6 ^c^	309.3 ± 4.2 ^a^
	Quercetin glycoside	148.6 ± 4.5 ^b^		73.1 ± 1.5 ^c^	297.7 ± 3.2 ^a^
	Quercetin	86.9 ± 6.0 ^b^		11.2 ± 0.1 ^c^	848.6 ± 14.7 ^a^
	Total phenols	11,351.6 ± 42.9 ^a^	1423.1 ± 36.5 ^c^	832.3 ± 0.2 ^d^	3854.2 ± 68.9 ^b^

Note: The lowercase letters situated adjacent to the standard deviation denote the separation of means with a confidence level of 95%.

**Table 4:** Average values of the antioxidant activity of the medicinal leaves.

		*Allium schoenoprasum*	*Cyclanthus bipartitus*	*Brugmansia candida* White	*Brugmansia candida* Pink
Antioxidant activity (mmol TE/100 g DW)					
	DPPH	1.7 ± 0.1 ^b^	1.0 + 0.1 ^c^	4.0 ± 0.5 ^a^	4.5 ± 0.4 ^a^
	ABTS	4.1 ± 1.5 ^a^	2.6 + 0.4 ^c^	2.7 ± 0.6 ^bc^	2.8 ± 0.4 ^b^

Note: The lowercase letters situated adjacent to the standard deviation denote the separation of means with a confidence level of 95%.

**Table 5:** Average values of the antimicrobial activity of the medicinal leaf extracts.

	Zone of Inhibition (mm)					
	Bacterial Strain				Fungal Strain	
Leaf extracts	*E. coli* ATCC 8739	*S. aureus* ATCC 6538P	*P. aeruginosa* ATCC 9027	*S. mutans* ATCC 25175	*C. albicans* ATCC 1031	*C. tropicalis* ATCC 13803
*Allium schoenoprasum*	-	-	-	-	-	-
*Cyclanthus bipartitus*	-	12.5 ± 0.7 ^a^	7.5 ± 0.7 ^b^	11.5 ± 0.7 ^a^	-	-
*Brugmansia candida* white	5.5 ± 0.5 ^c^	6.5 ± 0.7 ^b^	-	5.0 ± 0.3 ^c^	6.0 ± 0.5 ^b^	12.5 ± 0.5 ^a^
*Brugmansia candida* Pink	9.5 ± 2.1 ^b^	9.5 ± 2.1 ^b^	11.5 ± 0.5 ^a^	8.5 ± 1.0 ^c^	7.5 ± 0.5 ^c^	11.5 ± 0.7 ^a^
Control	26.2 ± 1.6 ^b^	22.5 ± 3.3 ^d^	25.0 ± 1.7 ^c^	31.1 ± 1.5 ^a^	10.6 ± 2.4 ^f^	16.8 ± 2.2 ^e^

Note: -, non-active at the tested concentrations. The lowercase letters situated adjacent to the standard deviation denote the separation of means with a confidence level of 95%.

**Table 6:** Minimal inhibitory concentration of the medicinal leaf extracts.

Microbial Strain	Minimal Inhibitory Concentration (mg/mL)		
	*C. bipartitus*	*B. candida* White	*B. Candida* Pink
*E. coli* ATCC 8739	-	41.7	10.6
*P. aeruginosa* ATCC 9027	125.0	-	21.1
*S. aureus* ATCC 6538P	31.3	41.7	10.6
*S. mutans* ATCC 25175	2.0	20.8	5.3
*C. albicans* ATCC 1031	-	41.7	42.2
*C. tropicalis* ATCC 13803	-	20.8	42.2

## Data Availability

The original contributions presented in this study are included in the article. Further inquiries can be directed to the corresponding author.
